# Perfusion Imaging for Endovascular Thrombectomy in Acute Ischemic Stroke Is Associated With Improved Functional Outcomes in the Early and Late Time Windows

**DOI:** 10.1161/STROKEAHA.121.038010

**Published:** 2022-05-04

**Authors:** Permesh Singh Dhillon, Waleed Butt, Anna Podlasek, Norman McConachie, Robert Lenthall, Sujit Nair, Luqman Malik, Thomas C. Booth, Pervinder Bhogal, Hegoda Levansri Dilrukshan Makalanda, Oliver Spooner, Alex Mortimer, Saleh Lamin, Swarupsinh Chavda, Han Seng Chew, Kurdow Nader, Samer Al-Ali, Benjamin Butler, Dilina Rajapakse, Jason P. Appleton, Kailash Krishnan, Nikola Sprigg, Aubrey Smith, Kyriakos Lobotesis, Phil White, Martin A. James, Philip M. Bath, Robert A. Dineen, Timothy J. England

**Affiliations:** Interventional Neuroradiology (P.S.D., N.M., R.L., S.N., L.M.), Queens Medical Centre, Nottingham University Hospitals NHS Trust, United Kingdom.; Stroke (K.K., N.S., P.M.B.), Queens Medical Centre, Nottingham University Hospitals NHS Trust, United Kingdom.; National Institute for Health and Care Research Nottingham Biomedical Research Centre (P.S.D., A.P., R.A.D.), University of Nottingham, United Kingdom.; Stroke Trials Unit, Mental Health and Clinical Neuroscience (N.S., P.M.B., T.J.E.), University of Nottingham, United Kingdom.; Radiological Sciences, Mental Health and Clinical Neuroscience (R.A.D.), University of Nottingham, United Kingdom.; Interventional Neuroradiology, Queen Elizabeth Hospital, University Hospitals Birmingham NHS Trust, United Kingdom (W.B., S.L., S.C., H.S.C., K.N., S.A.-A., B.B., D.R.).; Department of Neuroradiology, King’s College Hospital NHS Foundation Trust, London, United Kingdom (T.C.B.).; School of Biomedical Engineering and Imaging Sciences, King’s College London, United Kingdom (T.C.B.).; Interventional Neuroradiology (P.B., H.L.D.M.), The Royal London Hospital, Barts Health NHS Trust, United Kingdom.; Stroke (O.S.), The Royal London Hospital, Barts Health NHS Trust, United Kingdom.; Interventional Neuroradiology, Southmead Hospital, North Bristol NHS Trust, United Kingdom (A.M.).; Stroke, University Hospitals Birmingham NHS Foundation Trust, Edgbaston, United Kingdom (J.P.A.).; Institute of Applied Health Research, College of Dental and Medical Sciences, University of Birmingham, United Kingdom (J.P.A.).; Interventional Neuroradiology, Hull Royal Infirmary, Hull and East Yorkshire Hospitals NHS Trust, United Kingdom (A.S.).; Interventional Neuroradiology, Charing Cross Hospital, Imperial College Healthcare NHS Trust, London, United Kingdom (K.L.).; Translational and Clinical Research Institute, Faculty of Medical Sciences, Newcastle University and Newcastle upon Tyne Hospitals NHS Foundation Trust, United Kingdom (P.W.).; University of Exeter Medical School, United Kingdom (M.A.J.).; Royal Devon and Exeter NHS Foundation Trust, United Kingdom (M.A.J.).; Sentinel Stroke National Audit Programme, King’s College London, United Kingdom (M.A.J.).; Stroke, University Hospitals of Derby and Burton NHS Foundation Trust, United Kingdom (T.J.E.).

**Keywords:** computed tomography angiography, ischemia, neuroimaging, perfusion imaging, thrombectomy

## Abstract

The impact on clinical outcomes of patient selection using perfusion imaging for endovascular thrombectomy (EVT) in patients with acute ischemic stroke presenting beyond 6 hours from onset remains undetermined in routine clinical practice.

**Methods:**

Patients from a national stroke registry that underwent EVT selected with or without perfusion imaging (noncontrast computed tomography/computed tomography angiography) in the early (<6 hours) and late (6–24 hours) time windows, between October 2015 and March 2020, were compared. The primary outcome was the ordinal shift in the modified Rankin Scale score at hospital discharge. Other outcomes included functional independence (modified Rankin Scale score ≤2) and in-hospital mortality, symptomatic intracerebral hemorrhage, successful reperfusion (Thrombolysis in Cerebral Infarction score 2b–3), early neurological deterioration, futile recanalization (modified Rankin Scale score 4–6 despite successful reperfusion) and procedural time metrics. Multivariable analyses were performed, adjusted for age, sex, baseline stroke severity, prestroke disability, intravenous thrombolysis, mode of anesthesia (Model 1) and including EVT technique, balloon guide catheter, and center (Model 2).

**Results:**

We included 4249 patients, 3203 in the early window (593 with perfusion versus 2610 without perfusion) and 1046 in the late window (378 with perfusion versus 668 without perfusion). Within the late window, patients with perfusion imaging had a shift towards better functional outcome at discharge compared with those without perfusion imaging (adjusted common odds ratio [OR], 1.45 [95% CI, 1.16–1.83]; *P*=0.001). There was no significant difference in functional independence (29.3% with perfusion versus 24.8% without; *P*=0.210) or in the safety outcome measures of symptomatic intracerebral hemorrhage (*P*=0.53) and in-hospital mortality (10.6% with perfusion versus 14.3% without; *P*=0.053). In the early time window, patients with perfusion imaging had significantly improved odds of functional outcome (adjusted common OR, 1.51 [95% CI, 1.28–1.78]; *P*=0.0001) and functional independence (41.6% versus 33.6%, adjusted OR, 1.31 [95% CI, 1.08–1.59]; *P*=0.006). Perfusion imaging was associated with lower odds of futile recanalization in both time windows (late: adjusted OR, 0.70 [95% CI, 0.50–0.97]; *P*=0.034; early: adjusted OR, 0.80 [95% CI, 0.65–0.99]; *P*=0.047).

**Conclusions:**

In this real-world study, acquisition of perfusion imaging for EVT was associated with improvement in functional disability in the early and late time windows compared with nonperfusion neuroimaging. These indirect comparisons should be interpreted with caution while awaiting confirmatory data from prospective randomized trials.

Endovascular thrombectomy (EVT) for large vessel occlusion in acute ischemic stroke (AIS) is effective when initiated within 6 hours of stroke onset, although greater treatment benefit was observed when perfusion-based imaging was used for patient selection of limited core infarct volumes compared with nonperfusion based neuroimaging.^1–5^ The DEFUSE-3 (Endovascular Therapy Following Imaging Evaluation for Ischemic Stroke 3) and DAWN (Clinical Mismatch in the Triage of Wake Up and Late Presenting Strokes Undergoing Neurointervention With Trevo) trials only demonstrated benefit for patients presenting between 6 to 16 or 6 to 24 hours, respectively, from the onset of stroke or last known well, with a suitable infarct core-penumbra ratio or clinical deficit mismatch demonstrated by advanced neuroimaging (computed tomography [CT] perfusion or magnetic resonance [MR] imaging).^6,7^

However, perfusion imaging may exclude some patients who are also likely to benefit from EVT, and many institutions have limited access to urgent CT perfusion or MR imaging and so select patients for EVT on the basis of noncontrast CT (NCCT) and CT angiography (CTA). This practice results in potentially broader and more heterogeneous penumbra-core tissue characteristics compared with trial cohorts. There is paucity of data comparing patient outcomes based on the modality of imaging triage, particularly in the late treatment window (6–24 hours from stroke onset or last known well). Hence, using a large comprehensive national stroke registry, we sought to evaluate clinical outcomes following EVT in patients with AIS selected with or without perfusion imaging in the early (within 6 hours from stroke onset or last known well) and late time windows.

## Methods

### Ethics

The Sentinel Stroke National Audit Programme (SSNAP) has permission to collect patient data without explicit consent, granted by the Confidentiality Advisory Group of the National Health Service Health Research Authority under Section 251. Pseudonymized data use was approved by the Healthcare Quality Improvement Partnership Data Access Request Group. Additional ethical approval was not sought or required for this study.

### Data Source and Study Design

Data access requests should be directed to SSNAP as the data provider and Healthcare Quality Improvement Partnership as the data controller.

We performed a cohort study on prospectively collected data of patients enrolled in SSNAP according to the Strengthening the Reporting of Observational Studies in Epidemiology guidelines. SSNAP is a national stroke registry that includes all hospitals admitting patients presenting with acute stroke in England, Wales, and Northern Ireland (covering 92% of the population in the United Kingdom).^8^ Overall case ascertainment of SSNAP is estimated to be over 90% of all acute stroke admissions.^8^ Patient data, which include demographic and clinical characteristics, treatments, and outcomes, were submitted prospectively by clinical teams using a secure web-based case report form with real-time data validation checks to ensure data quality, from the time of admission up to 6 months after stroke.

Pseudonymized individual-level data of adult patients (≥18 years) presenting with AIS who received EVT between October 1, 2015 (inception of the EVT section of SSNAP) and March 31, 2020, in England and Wales were included. Patients were divided into 2 groups according to the time from onset of stroke, or last known well, to arterial puncture: (1) early window (<6 hours) and (2) late window (6–24 hours). Within each time window cohort, patients were further dichotomized according to the imaging selection modality for EVT eligibility: (1) NCCT with CT perfusion imaging±CTA and (2) NCCT±CTA. Patients treated beyond 24 hours from stroke onset or last known well and those with missing discharge modified Rankin Scale (mRS) data were excluded (complete case analysis performed). The selection of EVT-eligible patients was at the discretion of the practitioners based on each institution’s protocol.^9^ No specific limits were applied to the clinical inclusion criteria, including age, prestroke disability, and baseline stroke severity from the National Institutes of Health Stroke Scale (NIHSS). Data on the parenchymal imaging findings and clot location were not available in the registry.

### Outcome Measures

The primary outcome was function assessed using the mRS score at ultimate hospital discharge, ranging from 0, no symptoms to 5, severe disability/bedridden and 6, death. Other functional outcome measures were mRS score at 6 months, functional independence (mRS score ≤2) or excellent functional outcome (mRS score ≤1 or equal to the prestroke mRS) at hospital discharge and at 6 months, early neurological improvement (NIHSS score decrease ≥4 between admission and 24 hours or NIHSS score 0–1 at 24 hours), early neurological deterioration (24-hour NIHSS score increase ≥4 from baseline), and futile recanalization (mRS score 4–6) at hospital discharge or worsening of the prestroke disability of mRS score of 4–5 despite successful reperfusion (modified Thrombolysis in Cerebral Infarction score of 2b–3). Procedural outcomes were successful reperfusion and complete reperfusion (modified Thrombolysis in Cerebral Infarction score of 3) at the end of EVT.

Safety outcomes were in-hospital mortality, any type of intracranial hemorrhage (ICH) and symptomatic intracranial hemorrhage (sICH) defined according to ECASS (European Collaborative Acute Stroke Study) II^10^ as any ICH with NIHSS score of ≥4 within 24 hours or death. Workflow time metrics were stroke onset-to-arterial puncture, neuroimaging-to-arterial puncture, arterial puncture-to-first pass, and total procedural time, defined as arterial puncture-to-final reperfusion/angiogram. Functional outcome (mRS) was assessed by a member of the stroke team/physician at discharge or during a scheduled clinical visit at 6 months or by a specialist nurse during a follow-up telephone interview.

### Statistical Analysis

Study characteristics were summarized by early and late time windows using descriptive statistics for patient demographics, clinical characteristics and comorbidities, EVT technique and time metrics. Continuous variables were expressed as means and SD, and categorical variables were expressed as frequencies or percentages. Comparisons of baseline variables were made using the χ^2^, Mann-Whitney *U* test, or Student *t* test, wherever applicable.

Univariate analyses of the outcome measures used ordinal logistic regression for the full-scale mRS (main functional outcome) and binary regression analysis for the remaining dichotomized clinical outcomes. Multiple variable analysis was also conducted: Model 1 adjusted for variables of clinical relevance: age (5-year age bands <60, 60–64, 65–69, 70–74, 75–79, 80–84, 85–89, and >90 years), sex, baseline stroke severity (NIHSS), mode of anesthesia (local or general anesthesia, or conscious sedation), prestroke functional status (mRS) and prior administration of IV tPA (intravenous tissue-type plasminogen activator). Model 2 was adjusted for Model 1 and balloon guide catheter use, EVT technique, and center.

Analyses of binary and ordinal outcomes were expressed as an odds ratio (OR) with a 95% CI. Two subgroup analyses were performed involving: (1) only patients in centers that used either perfusion or nonperfusion imaging in the early and late windows (patients in centers that virtually always used perfusion imaging only or nonperfusion imaging only were excluded) and (2) those with futile recanalization versus those without. A sensitivity analysis was also performed, only accounting for patients with a witnessed stroke onset and excluding patients presenting with wake-up stroke or last known well. Two-tailed *P*<0.05 was considered statistically significant. All analyses were conducted using StataSE 16.1.

## Results

### Characteristics of Study Population

**Figure 1.**
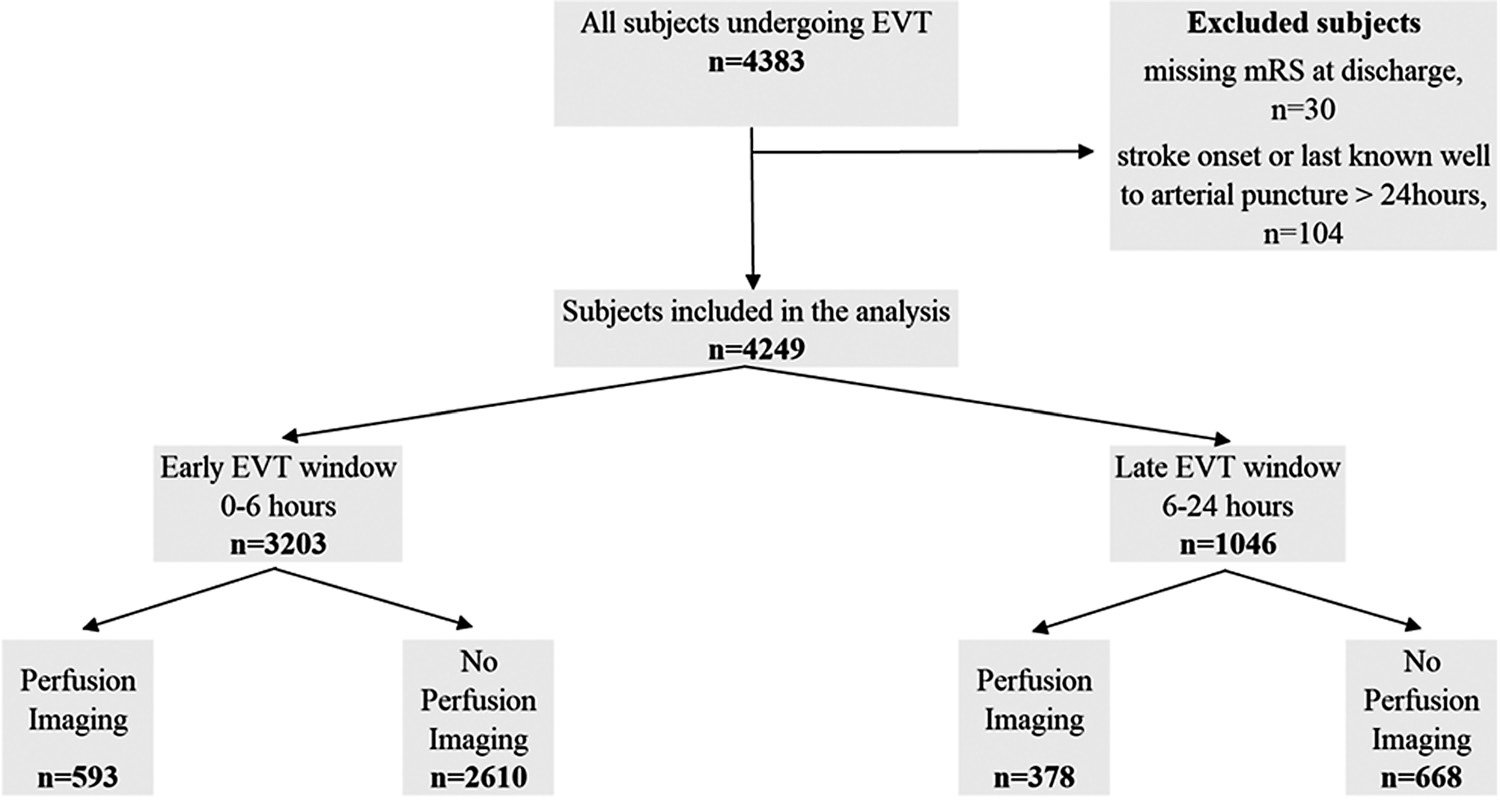
**Flow chart of the patient inclusion, exclusion, and outcome data for endovascular thrombectomy (EVT) treatment in the early (<6 h) and late (6–24 h) time windows, stratified according to the imaging modality acquisition (without or without perfusion imaging).** mRS indicates modified Rankin Scale; and n, number of events.

A total of 4383 patients originally admitted to 123 hospitals underwent EVT at 25 EVT-capable neuroscience centers during the study period. Of these, 104 patients treated beyond 24 hours and 30 patients with lack of data on the mRS score at discharge were excluded (Figure 1). We included 4249 patients (971 [22.9%] selected with and 3278 [77.1%] selected without perfusion imaging). A total of 3203 (75.4%) patients were treated within 6 hours (593 [18.5%] selected with and 2610 [81.5%] selected without perfusion imaging) while 1046 (24.6%) patients were treated between 6 and 24 hours from stroke onset or last known well (378 [36.1%] selected with and 668 [63.9%] selected without perfusion imaging; Figure 1). Overall, 197 patients had significant prestroke disability (mRS score 3–5) and 257 patients had an NIHSS score of <6 on admission. A total of 2807 patients (66.0%) had a documented precise time of stroke onset, the remainder were documented as last known well.

Compared with patients selected without perfusion imaging, those selected using perfusion imaging had a lower likelihood of receiving IV tPA in the late window (27.5% versus 33.8%; *P*=0.035, Table S1) but not in the early window (*P*=0.09, Table S2). In the late window, the baseline NIHSS scores were similar (perfusion: 16 [9–20] versus nonperfusion: 16 [10–21]; *P*=0.41, Table S1), but patients selected with perfusion imaging in the early window had lower baseline NIHSS scores compared with those selected without perfusion (17 [12–21] versus 18 [13–22]; *P*=0.015, Table S2). In both time windows, patients selected with perfusion imaging were more likely to be treated using a combined technique of stent-retriever and thromboaspiration (Tables S1 and S2). No significant differences were observed between CT perfusion and non-CT perfusion groups in both time windows for the remaining baseline characteristics (Tables S1 and S2).

The mean onset-to-treatment in the perfusion imaging group was significantly longer in the late time window compared with the nonperfusion group (671.1±251.5 versus 619.5±247.5 minutes; Table S1) while no difference was seen in the early window (perfusion: 229.6±68.4 versus nonperfusion: 232.5±67.4 minutes; Table S2). In the late window, neuroimaging-to-arterial puncture time was similar (perfusion: 158.0±169.8 versus nonperfusion: 160.3±189.4 minutes), while the door-to-end of procedure time (325.5±219.1 versus 334.9±216.3 minutes) and procedural time (54.4±35.9 versus 61.9±42.4 minutes) were shorter in the perfusion imaging group compared with the nonperfusion group (Table S1). In the early window, no significant differences were identified in the neuroimaging-to-arterial puncture time (*P*=0.51) or procedural time (*P*=0.06) between imaging groups (Table S2). The distribution of patients relative to onset-to-puncture time across both time windows is presented in Figure S1.

### Outcomes

#### Perfusion Imaging Versus Nonperfusion Imaging in the Late and Early Time Windows

**Figure 2.**
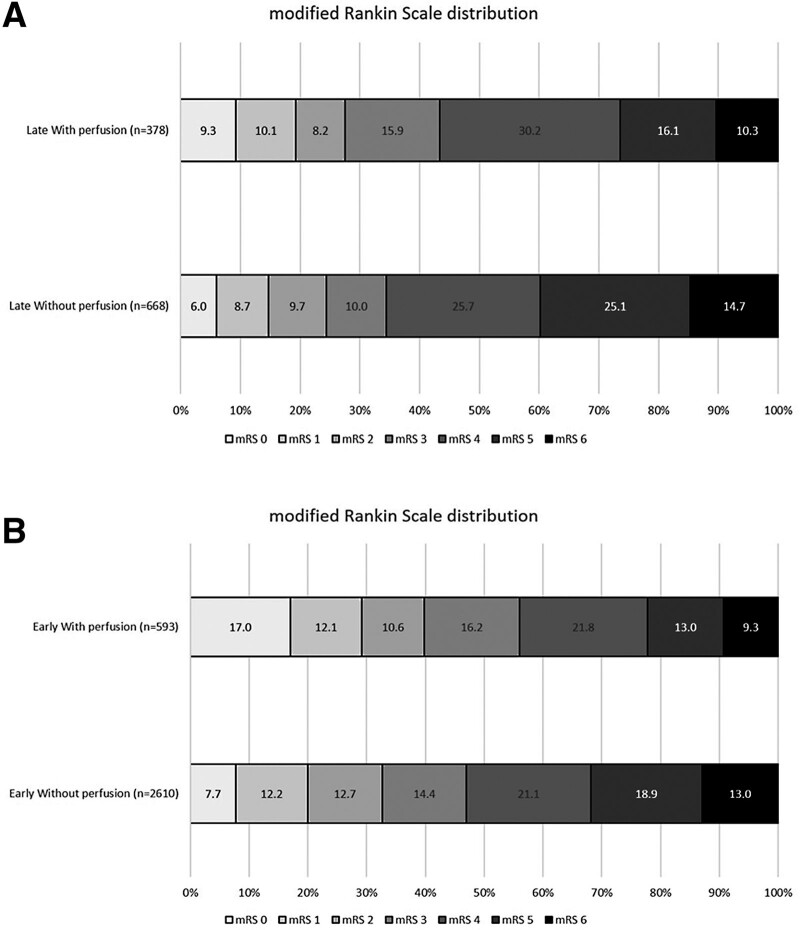
**Distribution of the modified Rankin Scale (0, no disability to 5, severe disability and 6, death) at discharge.** Endovascular thrombectomy (EVT) treatment in patients selected with or without perfusion imaging in (**A**) the late window (6–24 h from stroke onset or last known well) and (**B**) early window (within 6 h from stroke onset or last known well).

Within the late window, selection using perfusion imaging was associated with a significantly higher odds of improving the mRS score by 1 point on the mRS scale at discharge (Figure 2, Table 1; adjusted common OR, 1.45 [95% CI, 1.16–1.83]; *P*=0.001) compared with those selected without perfusion imaging. Perfusion imaging was associated with lower odds of futile recanalization (53.7% [with perfusion] versus 60.4% [without], Model 2: adjusted OR [aOR], 0.70 [95% CI, 0.50–0.97]; *P*=0.034). There was no significant difference between imaging selection modalities in functional independence (mRS score ≤2) at discharge (29.3% [with perfusion] versus 24.8% [without]; *P*=0.210), and safety outcome measures of sICH (3.3% [with perfusion] versus 4.6% [without]; *P*=0.531) and in-hospital mortality (10.6% [with perfusion] versus 14.3% [without]; *P*=0.053). Similar associations were observed even after adjusting for additional imbalanced variables (Model 2) in the early and late time windows (Tables 1 and 2).

**Table 1.**
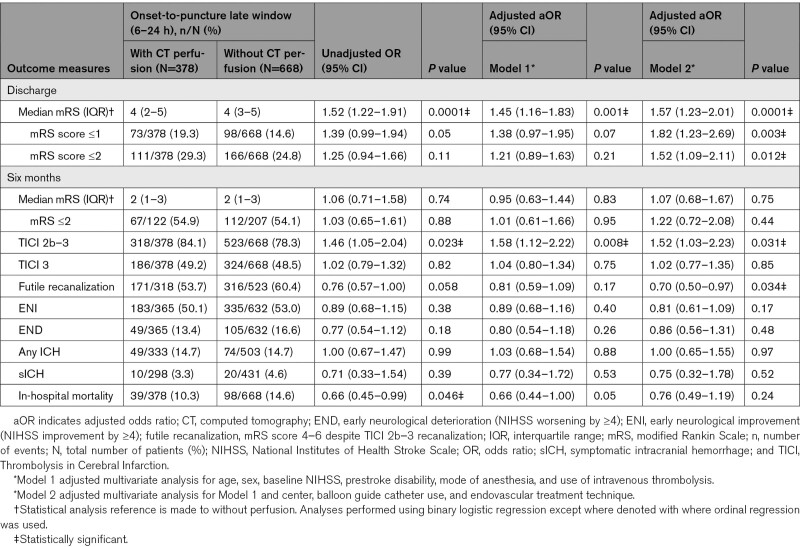
Table of Outcomes Dichotomized by Imaging Modality Selection of Perfusion Versus Without Perfusion Imaging in the Late Time Window (6–24 Hours From Stroke Onset or Last Known Well to Endovascular Treatment)

**Table 2.**
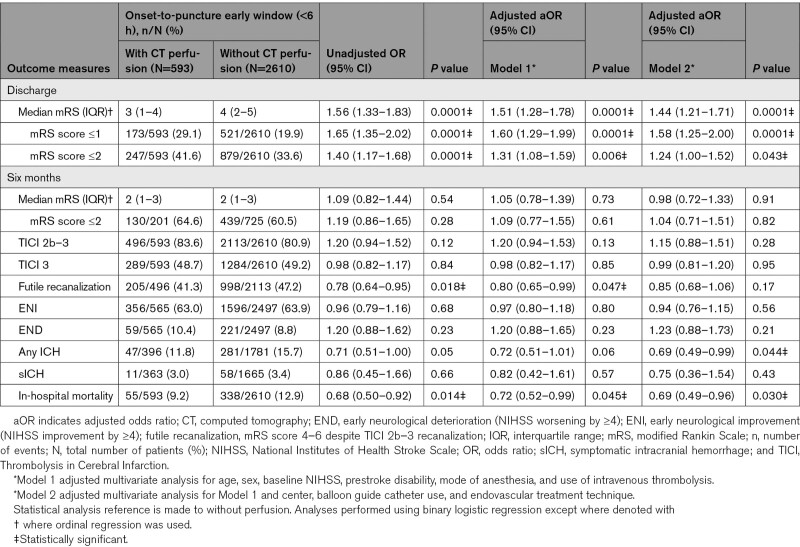
Table of Outcomes Dichotomized by Imaging Modality Selection of Perfusion Versus Without Perfusion Imaging in the Early Time Window (Within 6 Hours From Stroke Onset or Last Known Well to Endovascular Treatment)

In the early window, compared with those selected without perfusion imaging, selection with perfusion imaging was associated with a significantly improved odds of a better functional outcome (ordinal mRS shift: Figure 2, Table 2; adjusted common OR, 1.51 [95% CI, 1.28–1.78]; *P*=0.0001) and functional independence (mRS score ≤2) at discharge (41.6% versus 33.6%; aOR, 1.31 [95% CI, 1.08–1.59]; *P*=0.006), while the odds of in-hospital mortality (9.2% versus 12.9%; aOR, 0.72 [95% CI, 0.52–0.99]; *P*=0.045) and futile recanalization (Model 1: aOR, 0.80 [95% CI, 0.65–0.99]; *P*=0.047) were reduced.

Sensitivity analysis of patients in the late window with a witnessed stroke onset only and selected using perfusion imaging demonstrated improved odds of improving the mRS score at discharge (ordinal shift: Table S3; adjusted common OR, 1.55 [95% CI, 1.16–2.06]; *P*=0.002). Patients selected with perfusion imaging at centers that employed a mix of either perfusion or nonperfusion imaging in the early window had lower rates of sICH compared with those selected without (Model 2; aOR, 0.36 [95% CI, 0.13–0.95]; *P*=0.04; Table S4). No other significant associations were identified in the remaining outcomes in the subgroups across both time windows (Tables S4 and S5). Baseline characteristics of patients treated at centers that used perfusion imaging only (early window: 9.7%, late window: 3.7%), nonperfusion imaging only (early window: 51.6%, late window: 41.7%), or a mix of both (early window: 38.6%, late window: 54.4%), are demonstrated in Table S6. The subgroup of patients with futile recanalization (early window: 46.1%, late window: 57.9%) had a significantly higher baseline NIHSS score versus those without in the late window (12 [7–18] versus 17 [11–22]; *P*=0.001), with a similar difference also noted in the early window (Table S7). There was a significantly higher rate of sICH among patients with futile recanalization versus those without in both the late (5.2% versus 0.7%) and early (5.3% versus 0.6%) windows (Table S7).

## Discussion

This exploratory study of 4249 patients from a national stroke registry provides real-world comparative data on clinical outcomes following EVT in patients selected with and without perfusion imaging in the late (6–24 hours from stroke onset or last known well) and early (<6 hours) time windows. Compared with patients selected without perfusion imaging, improved functional outcome was observed at discharge in patients selected with perfusion imaging in both late and early windows. Perfusion-based selection was also significantly associated with reduced futile recanalization in both time windows and in-hospital mortality in the early window. Successful reperfusion rates were higher in patients selected with perfusion imaging in the late window but not in the early window. Safety outcomes, including sICH and early neurological deterioration, were similar across both groups.

These findings contrast with previous studies that demonstrated no functional improvement associated with patient selection using CT perfusion compared with nonperfusion imaging selection in the late window.^11–13^ These studies were either of a modest sample size and/or were limited to patients that broadly mirrored the inclusion criteria of the DAWN or DEFUSE-3 randomized clinical trials (RCTs).^11–13^ Our large cohort of patients included those not conforming to the strict clinical criteria of these RCTs, thereby improving generalizability in our cohort study. Although increased radiation exposure and potential treatment delays have been associated with CT perfusion imaging acquisition,^14^ there was minimal difference in the mean neuroimaging-to-arterial puncture times in our cohort across both EVT windows. Nonetheless, the results from the present study should be interpreted with caution when considering the optimum imaging selection paradigm at a population level due to the denominator fallacy.^15^ For example, the yield of improved functional outcomes by super-selecting patients with more favorable target-mismatch profiles may be at the expense of excluding patients with a broader range of tissue characteristics who could still potentially benefit from the EVT treatment effect.^16^

Based on the results of DAWN and DEFUSE-3, current American Heart Association/American Stroke Association guidelines suggest the use of advanced neuroimaging (CT perfusion, diffusion-weighted MR, or MR perfusion) and strict criteria matching the RCTs’ eligibility for patient selection for EVT beyond 6 hours from stroke onset.^17^ Adherence to such recommendations is impeded in many parts of the world by resource constraints and limited access to urgent advanced imaging. Routine clinical practice in the UK differs from the clinical trial setting and other developed nations delivering EVT, as many institutions use NCCT/CTA (without CT perfusion or MR imaging) to visually estimate the core infarct size (ASPECTS [Alberta Stroke Program Early CT Score]) and collateral supply in both the early and late time windows. Given the large positive treatment effect observed in the late-window RCTs, it is plausible that there is a net treatment benefit in patients selected without perfusion imaging.^13^ While awaiting results from more inclusive RCTs,^18,19^ it is reasonable to maintain a pragmatic patient-tailored clinical approach to maximizing the potential benefits of EVT for late-presenting AIS patients.

In the early window, our findings are consistent with previous meta-analyses of primarily early window studies that reported increased odds of favorable outcomes following EVT in patients selected with perfusion imaging.^2,20^ In contrast, pooled analysis of the HERMES (Highly Effective Reperfusion Using Multiple Endovascular Devices) data of patients treated with EVT suggested that core-penumbra mismatch ratio assessed by CT perfusion was not independently associated with improved outcomes.^21^ These differences may, in part, be explained by study design and patient selection, as well as the lack of patients without mismatch in the HERMES cohort. A recent study also suggested that patients with a completed infarct based on perfusion imaging in the early window were more likely to have futile recanalization.^22^ Overall, a CT perfusion-based imaging paradigm in the early window is similarly subject to denominator bias and is not mandated based on current guidelines.^17^

The role of initial imaging modality on futile recanalization has recently been investigated in a study which reported that MR imaging (compared with CT perfusion) was associated with a decreased risk of futile recanalization.^23^ However, to our knowledge, no studies have reported on the association of futile recanalization when comparing CT perfusion to nonperfusion imaging (NCCT/CTA). It is plausible that the higher rates of futile recanalization in our nonperfusion imaging cohort may be explained by potentially more generous inclusion of patients with larger ischemic core volumes, indirectly illustrated by the higher baseline stroke severity NIHSS scores among patients with futile recanalization. Although higher rates of sICH and early neurological deterioration might be expected in the nonperfusion group using this assumption, we found no difference in these safety outcome measures.

The strengths of this study include its large sample size, the national coverage of a diverse range of hospitals and EVT-capable neuroscience centers (generalizability), and the high case ascertainment with consecutive patient enrollment. The accuracy and high-quality data within the SSNAP database results from standardized case definitions and coding instructions, internal validation, audit trails, and regular data quality reports for all participating sites.^8^

There are several limitations in this study. First, due to its observational design, confounding by indication and selection bias may have influenced the results. Specifically, the use of perfusion imaging may have been chosen, consciously or subconsciously, on the basis of patient characteristics. Furthermore, the specific criteria used to select eligible AIS patients, and the number of patients who were excluded from EVT treatment according to the imaging selection modality were not available in our registry. Second, there was some missing data for certain outcome measures, including the mRS at 6 months. However, near-complete data (99.3%) were available for the primary outcome measure of mRS at discharge and previous studies have shown that functional outcomes at hospital discharge correlate highly with functional outcomes at 3 months.^24^ Third, unaccounted variables such as the lack of the ASPECTS, collateral status, clot location, or infarct volumes, all of which are covariates of interest in patient selection were not available in the registry. However, direct comparisons would not be feasible between groups due to the variability in infarct volume estimation across perfusion and nonperfusion imaging modalities.^25^ Furthermore, the lack of standardization of imaging protocols and postprocessing software across institutions, potentially heterogeneous penumbra-core tissue characteristics present in our cohort and a likely lower clinical threshold for offering EVT employed in routine practice may account for some of the differences observed. Fourth, there were some differences in between-group baseline characteristics. However, key clinical variables were adjusted for in the multivariable analyses, and only intravenous thrombolysis was associated with better functional outcome in the early window, although statistical adjustment is known not to address large differences in covariates. Although no significant associations in the functional outcomes were identified in the subgroup analysis of patients treated at centers that utilize a mix of perfusion and nonperfusion imaging across both time windows, it is possible that some of these analyses were underpowered. Fifth, a proportion of patients included in our study presented with the best-estimated onset of stroke (last known well), which may have overestimated the time since stroke onset. However, similar associations with the outcomes remained in our sensitivity analysis of patients with a witnessed stroke onset. Sixth, lack of available data on transferred patients precluded analysis on potential differences in outcomes. Last, the outcome measures, including the angiographic outcomes of vessel reperfusion, were self-assessed rather than independently evaluated by a core laboratory.

## Conclusions

In this large real-world study, acquisition of perfusion imaging for EVT was associated with improvement in functional outcome in the early and late time windows compared with nonperfusion imaging. These indirect comparisons should be interpreted with caution while awaiting confirmatory data from more inclusive prospective randomized trials, which may prove that a net treatment benefit may still be sustained in patient groups selected without perfusion imaging in the late window.

## Article Information

### Acknowledgments

We thank all individuals and organizations participating in the Sentinel Stroke National Audit Programme (SSNAP).

### Sources of Funding

The Sentinel Stroke National Audit Programme (SSNAP) is commissioned by the Health Quality Improvement Partnership and funded by National Health Service (NHS) England and the Welsh Government. Dr James is supported by the National Institute for Health Research Applied Research Collaboration South West Peninsula. Dr Booth is supported by the Wellcome/EPSRC Centre for Medical Engineering [WT 203148/Z/16/Z]. This research was funded in whole, or in part, by the Wellcome Trust [WT 203148/Z/16/Z]. For the purpose of open access, the author has applied a CC BY public copyright licence to any Author Accepted Manuscript version arising from this submission. The views expressed in this publication are those of the author(s) and not necessarily those of the National Institute for Health Research or the Department of Health and Social Care. No specific funding was sought for this study.

### Disclosures

Dr Bhogal reports travel support from Perflow; compensation from Cerenovus for consultant services; compensation from Balt USA, LLC for consultant services; compensation from Vesalio for consultant services; compensation from phenox Inc for consultant services; and compensation from Brainomix for consultant services. Dr Makalanda reports compensation from Cerenovus for consultant services; compensation from Balt USA, Perfuze for consultant services; compensation from Stryker for consultant services; compensation from Penumbra for consultant services; compensation from Microvention for consultant services; compensation from Neuroventures for consultant services; and compensation from Brainomix for consultant services. Dr Spooner reports compensation from Brainomix for consultant services. Dr Appleton reports grants from National Institute for Health Research. Dr White reports grants from Penumbra, Inc to other; grants from MicroVention, Inc to other; grants from Stryker to other; compensation from MicroVention, Inc for consultant services; and grants from Medtronic to other. Dr James reports compensation from Medtronic for consultant services and grants from Healthcare Quality Improvement Partnership to other. P.M. Bath is Stroke Association Professor of Stroke Medicine, an Emeritus National Institute for Health and Care Research Senior Investigator and reports compensation from Phagenesis for consultant services; compensation from DiaMedica for consultant services; and compensation from Moleac for consultant services. The other authors report no conflicts.

### Supplemental Material

Tables S1–S7

Figure S1

STROBE checklist

## Supplementary Material


