# Supplementary material for: Perfusion Imaging for Endovascular Thrombectomy in Acute Ischemic Stroke Is Associated With Improved Functional Outcomes in the Early and Late Time Windows
Source: Stroke. 2022 May 4;53(9):2770–8. doi: 10.1161/STROKEAHA.121.038010 (PMC9389941; doi:10.1161/STROKEAHA.121.038010)
Supplement: Supplementary file 1 [file str-53-2770-s001.pdf]

SUPPLEMENTAL MATERIAL

Table S1: Table of characteristics according to time from stroke onset or last known well to endovascular treatment among patients selected with or without perfusion imaging in the **late (6 to 24 hours) time window**.

Feature	Perfusion n (%) median (IQR) or mean±SD	Without Perfusion n (%) median (IQR) or mean±SD	P value
**Socio-demographics**			
Sample size	378	668	
Sex (male)	188 (49.7)	366 (54.8)	0.11
<60 years	102 (27.0)	220 (32.9)	
60-69	69 (18.3)	128 (19.2)	
70-79	118 (31.2)	177 (26.5)	0.17
80-89	77 (20.4)	130 (19.5)	
>90 years	12 (3.2)	13 (1.9)	
**Baseline characteristics**			
NIHSS on admission	16 (10-21)	16 (9-20)	0.41
Pre-stroke disability (mRS)	0 (0-1)	0 (0-1)	0.33
IV Thrombolysis	104 (27.5)	226 (33.8)	**0.035**
Thromboaspiration[∂]	85 (24.1)	180 (30.4)	0.11
Stent-retriever[∂]	33 (9.3)	112 (18.9)	**0.001**
Thromboaspiration & Stent-retriever[∂]	235 (66.5)	300 (50.7)	**0.001**
Proximal Balloon Flow Arrest	133 (35.2)	139 (20.8)	**0.001**
**Co-morbidities**			
Hypertension	185 (48.9)	304 (45.5)	0.28
Diabetes	55 (14.6)	85 (12.7)	0.40
Atrial fibrillation	71 (18.8)	137 (20.5)	0.50
Prior Stroke/TIA	50 (13.2)	96 (14.4)	0.60
Congestive heart failure	24 (6.4)	33 (4.9)	0.33
**Time metrics (mins)**			
Onset to Groin Puncture	671.9±251.5	619.3±247.5	**0.001**
Neuroimaging to Arterial Puncture	158.0±169.8	160.3±189.0	0.84
Arterial Puncture to First deployment	24.5±17.2	28.1±22.2	**0.008**
Arterial Puncture to End of Procedure	54.4±35.9	61.9±42.4	**0.004**
Door to End of Procedure	325.5±219.1	334.9±216.3	**0.001**

n = number of events, N = number of patients, SD = standard deviation, mRS = modified Rankin scale, TIA = transient ischemic attack, NIHSS = National Institutes of Health Stroke Scale TICI = thrombolysis in cerebral infarction, IV = intravenous. Groups are compared using Chi-squared and Mann-WhitneyU tests as appropriate. [∂] N=353 perfusion group, n= 592 without perfusion group

Table S2: Table of characteristics according to time from stroke onset or last known well to endovascular treatment among patients selected with or without perfusion imaging in the **early (<6 hours) time window**.

Feature	Perfusion n (%) median (IQR) or mean±SD	Without Perfusion n (%) median (IQR) or mean±SD	P value
**Socio-demographics**			
Sample size	593	2610	
Sex (male)	306 (51.6)	1450 (55.6)	0.08
<60 years	144 (24.3)	646 (24.8)	
60-69	117 (19.7)	534 (20.5)	
70-79	187 (31.5)	785 (30.1)	0.93
80-89	124 (20.9)	573 (21.9)	
>90 years	21 (3.5)	72 (2.8)	
**Baseline characteristics**			
NIHSS on admission	17 (12-21)	18 (13-22)	**0.031**
Pre-stroke disability (mRS)	0 (0-1)	0 (0-1)	**0.047**
IV Thrombolysis	382 (64.4)	1835 (70.3)	**0.005**
Thromboaspiration[∂]	116 (21.1)	892 (38.8)	**0.001**
Stent-retriever[∂]	89 (16.2)	436 (18.9)	0.31
Thromboaspiration & Stent-retriever[∂]	344 (62.6)	969 (42.1)	**0.001**
Proximal Balloon Flow Arrest	190 (32.0)	477 (18.3)	**0.001**
**Co-morbidities**			
Hypertension	295 (49.8)	1242 (47.6)	0.34
Diabetes	86 (14.5)	367 (14.1)	0.78
Atrial fibrillation	153 (25.8)	567 (21.7)	0.22
Prior Stroke/TIA	97 (16.4)	416 (15.9)	0.99
Congestive heart failure	41 (6.9)	121 (4.6)	0.26
**Time metrics (mins)**			
Onset to Arterial Puncture	229.6±68.4	232.5±67.4	0.34
Neuroimaging to Arterial Puncture	167.8±183.1	162.3±182.1	0.51
Arterial Puncture to First deployment	24.1±18.2	25.7±19.0	0.06
Arterial Puncture to End of Procedure	54.2±35.6	57.3±37.4	0.06
Door to End of Procedure	180.9±71.8	198.6±72.6	**0.001**

n = number of events, N = number of patients, SD = standard deviation, mRS = modified Rankin scale, TIA = transient ischemic attack, NIHSS = National Institutes of Health Stroke Scale TICI = thrombolysis in cerebral infarction, IV = intravenous. Groups are compared using Chi-squared and Mann-Whitney Utests as appropriate. [∂] n=549 perfusion group, n= 2297 without perfusion group

Table S3: Table of outcomes dichotomized by imaging modality selection of perfusion versus without perfusion imaging in patients that underwent endovascular thrombectomy in the late time window (6-24 hours from **witnessed stroke onset only**).

**Outcome measures**	**Onset To Puncture Late Window (6-24 hours) n/N (%)**		**Unadjusted OR (95% CI)**	**P value**	**Adjusted aOR (95% CI) Model 1****	**P value**	**Adjusted aOR (95% CI) Model 2****	**P value**
	**With CT Perfusion (**N=241)	**Without CT Perfusion** (N=454)						
Discharge								
Median mRS (IQR) †	4 (2-5)	4 (3-5)	1.49 (1.13 – 1.97)	**0.004***	1.55 (1.16 – 2.06)	**0.002***	1.78 (1.31 – 2.42)	**0.0001***
mRS ≤1	45/241 (18.6)	68/454 (14.9)	1.30 (0.86 – 1.97)	0.21	1.47 (0.95 – 2.29)	0.08	2.38 (1.43 – 3.96)	**0.001***
mRS ≤2	70/241 (29.0)	113/454 (24.8)	1.25 (0.87 – 1.75)	0.23	1.28 (0.87 – 1.86)	0.19	1.72 (1.13 – 2.61)	**0.010***
6 months								
Median mRS (IQR) †	3 (1-4)	2 (1-3)	0.91 (0.54 – 1.51)	0.72	0.97 (0.57 – 1.66)	0.92	1.06 (0.59 – 1.87)	0.84
mRS ≤2	34/70 (48.5)	75/142 (52.8)	0.84 (0.47 – 1.49)	0.56	0.93 (0.49 – 1.76)	0.83	1.13 (0.58 – 2.21)	0.70
TICI 2b-3	198/241 (82.1)	352/454 (77.5)	1.33 (0.89 – 1.98)	0.15	1.48 (0.98 – 2.24)	0.05	1.57 (0.98 – 2.51)	0.058
TICI 3	118/241 (48.9)	209/454 (46.0)	1.12 (0.82 – 1.53)	0.46	1.15 (0.83 – 1.60)	0.37	1.14 (0.80 – 1.62)	0.45
Futile Recanalization	131/241 (54.3)	297/454 (65.4)	0.63 (0.45 – 0.86)	**0.004***	0.61 (0.43 – 0.86)	**0.005***	0.58 (0.38 – 0.88)	**0.01***
ENI	110/233 (47.2)	218/429 (50.8)	0.86 (0.62 – 1.19)	0.37	0.85 (0.61 – 1.19)	0.37	0.77 (0.53 – 1.10)	0.15
END	28/233 (12.0)	65/429 (15.1)	0.76 (0.47 – 1.22)	0.26	0.78 (0.47 – 1.28)	0.33	0.83 (0.48 – 1.45)	0.53
Any ICH	31/209 (14.8)	46/330 (13.9)	1.07 (0.65 – 1.75)	0.77	1.16 (0.68 – 1.95)	0.57	1.16 (0.66 – 2.03)	0.58
sICH	5/189 (2.6)	11/283 (3.8)	0.67 (0.22 – 1.96)	0.46	0.62 (0.19 – 1.97)	0.42	0.69 (0.19 – 2.43)	0.56
In-hospital mortality	28/241 (11.6)	62/454 (13.6)	0.83 (0.51 – 1.33)	0.44	0.74 (0.44 – 1.22)	0.24	0.85 (0.49 – 1.47)	0.57

n = number of events / total number of patients (%), N = number of patients, aOR = adjusted odds ratio, CI = confidence interval, mRS = modified Rankin scale, sICH = symptomatic intracranial haemorrhage, TICI = thrombolysis in cerebral infarction, Futile Recanalization = mRS4-6 despite TICI2b-3 recanalization, ENI = Early neurological improvement (NIHSS improvement by ≥4), END= Early neurological deterioration (NIHSS worsening by ≥4). * = statistically significant **Model 1 adjusted multivariate analysis for age, sex, baseline NIHSS, pre-stroke disability, mode of anesthesia and use of intravenous thrombolysis. **Model 2 adjusted multivariate analysis for Model 1 and centre, balloon guide catheter use and endovascular treatment technique. Statistical analysis reference is made to 'without perfusion'. Analyses performed using binary logistic regression except where denoted with † where ordinal regression was used.

Table S4: Table of characteristics and outcomes according to time from stroke onset or last known well to endovascular treatment involving subgroup of patients in centres that utilised either perfusion or non-perfusion imaging in the early (< 6 hours) time window (patients in centres that virtually always utilised perfusion imaging only or non-perfusion imaging only were excluded).

**Feature**	**Perfusion n/N (%) median (IQR)**	**Without Perfusion n/N (%) median (IQR)**	**Adjusted aOR (95% CI) Model 1****	**P value**	**Adjusted aOR (95% CI) Model 2****	**P value**
**Sample size**	466	771	-	-	-	-
**Baseline characteristics**						
NIHSS on admission	18 (12-21)	18 (13-22)	-	**0.44**	-	-
Pre-stroke disability (mRS)	0 (0-1)	0 (0-1)	-	0.58	-	-
IV Thrombolysis	308 (66.1)	542 (70.3)	-	0.12	-	-
Thromboaspiration[∂]	78 (17.8)	293 (41.8)	-	**0.001**	-	-
Stent-retriever[∂]	76 (17.3)	102 (14.5)	-	0.13	-	-
Thromboaspiration & Stent-retriever[∂]	284 (64.8)	305 (43.5)	-	**0.001**	-	-
Proximal Balloon Flow Arrest	160 (34.3)	160 (20.7)	-	**0.001**	-	-
**Outcomes**						
mRS at discharge	3 (1-4)	3 (2-5)	1.22 (0.99-1.51)	0.052	1.18 (0.94-1.47)	0.14
mRS ≤2 at discharge	179/466 (38.4)	268/771 (34.7)	1.13 (0.88-1.45)	0.33	1.09 (0.82-1.43)	0.53
sICH	4/293 (1.3)	23/404 (5.6)	0.53 (0.22-1.22)	0.13	0.36 (0.13-0.95)	**0.04***
In-hospital mortality	43/466 (9.2)	84/771 (10.9)	0.82 (0.54-1.24)	0.35	0.78 (0.49-1.22)	0.28
Futile Recanalization	213/466 (45.7)	372/771 (48.2)	0.98 (0.75-1.28)	0.90	1.02 (0.76-1.36)	0.88

n = number of events, N = number of patients, mRS = modified Rankin scale, TIA = transient ischemic attack, NIHSS = National Institutes of Health Stroke Scale, IV = intravenous. Groups are compared using Chi-squared and Mann-Whitney U-tests as appropriate. [∂] n=438 perfusion group, n=700 non-perfusion group, futile recanalization=mRS 4-6 at despite TICI2b-3 recanalization, sICH = symptomatic intracranial haemorrhage, END= Early neurological deterioration (NIHSS worsening by ≥4). * = statistically significant **Model 1 adjusted multivariate analysis for age, sex, baseline NIHSS, pre-stroke disability, mode of anaesthesia, and use of intravenous thrombolysis. **Model 2 adjusted multivariate analysis for Model 1 and balloon guide catheter use and endovascular treatment technique.

Table S5: Table of characteristics and outcomes according to time from stroke onset or last known well to endovascular treatment involving subgroup of patients in centres that utilised either perfusion or non-perfusion imaging in the late (6-24 hours) time window (patients in centres that virtually always utilised perfusion imaging only or non-perfusion imaging only were excluded).

**Feature**	**Perfusion n/N (%) median (IQR)**	**Without Perfusion n/N (%) median (IQR)**	**Adjusted aOR (95% CI) Model 1****	**P value**	**Adjusted aOR (95% CI) Model 2****	**P value**
**Sample size**	312	258	-	-	-	-
**Baseline characteristics**						
NIHSS on admission	16 (10-20)	15 (8-20)	-	0.88	-	-
Pre-stroke disability (mRS)	0 (0-1)	0 (0-1)	-	0.46	-	-
IV Thrombolysis	86 (27.5)	75 (29.0)	-	0.69	-	-
Thromboaspiration[∂]	64 (21.7)	73 (30.2)	-	**0.03**	-	-
Stent-retriever[∂]	31 (10.5)	44 (18.2)	-	**0.012**	-	-
Thromboaspiration & Stent-retriever[∂]	199 (67.6)	124 (51.4)	-	**0.001**	-	-
Proximal Balloon Flow Arrest	120 (38.4)	52 (20.8)	-	**0.001**	-	-
**Outcomes**						
mRS at discharge	4 (2-5)	4 (2-5)	1.06 (0.78-1.43)	0.69	1.26 (0.91-1.74)	0.15
mRS ≤2 at discharge	86/312 (27.5)	76/258 (29.4)	0.88 (0.59-1.30)	0.53	1.27 (0.82-1.99)	0.27
sICH	9/243 (3.7)	6/157 (3.8)	0.98 (0.30-3.18)	0.97	0.96 (0.27-3.34)	0.95
In-hospital mortality	31/312 (9.9)	29/258 (11.2)	0.80 (0.45-1.41)	0.49	0.94 (0.51-1.75)	0.86
Futile Recanalization	181/312 (58.0)	153/258 (59.3)	1.01 (0.68-1.50)	0.95	0.80 (0.52-1.23)	0.33

n = number of events, N = number of patients, mRS = modified Rankin scale, TIA = transient ischemic attack, NIHSS = National Institutes of Health Stroke Scale, IV = intravenous. Groups are compared using Chi-squared and Mann-Whitney U-tests as appropriate. [∂] n=294 perfusion group, n=241 non-perfusion group, futile recanalization=mRS 4-6 at despite TICI2b-3 recanalization, sICH = symptomatic intracranial haemorrhage, END= Early neurological deterioration (NIHSS worsening by ≥4). * = statistically significant **Model 1 adjusted multivariate analysis for age, sex, baseline NIHSS, pre-stroke disability, mode of anaesthesia, and use of intravenous thrombolysis. **Model 2 adjusted multivariate analysis for Model 1 and balloon guide catheter use and endovascular treatment technique.

Table S6: Table of characteristics among patients selected for endovascular stroke treatment at centres that employ perfusion imaging only, non-perfusion imaging only, or a mix of either perfusion or non-perfusion imaging in the **early (< 6 hours)** and **late (6-24 hours) time windows.**

**Feature**	**Perfusion centres only n(%) median (IQR)**		**Non-Perfusion centres only n (%) median (IQR)**		**Mixed Perfusion Centres n (%) median (IQR)**	
	**Early**	**Late**	**Early**	**Late**	**Early**	**Late**
**Sample size**	313	39	1653	437	1237	570
**Baseline characteristics**						
NIHSS on admission	17 (12-21)	16 (7-18.5)	18 (13-22)	16 (9-21)	18 (12-22)	16 (9-20)
Pre-stroke disability (mRS)	0 (0-1)	0 (0-0.5)	0 (0-1)	0 (0-1)	0 (0-1)	0 (0-1)
IV Thrombolysis	214 (68.3)	8 (20.5)	1153 (69.7)	161 (36.8)	850 (68.7)	161 (28.2)
Thromboaspiration[∂]	115 (43.2)	13 (33.3)	522 (36.1)	117 (31.2)	371 (32.6)	137 (25.6)
Stent-retriever[∂]	42 (15.7)	2 (5.1)	305 (21.1)	69 (18.4)	178 (15.6)	75 (13.1)
Thromboaspiration & Stent-retriever[∂]	109 (40.9)	24 (61.5)	615 (42.6)	189 (50.4)	589 (51.7)	323 (56.6)
Proximal Balloon Flow Arrest	27 (8.6)	9 (23.1)	320 (19.3)	91 (20.8)	320 (25.8)	172 (30.2)

n = number of events, N = number of patients, mRS = modified Rankin scale, NIHSS = National Institutes of Health Stroke Scale, IV = intravenous. Groups are compared using Chi-squared and Mann-Whitney U-tests as appropriate. [∂] Early window: N=266 perfusion centres only group, n=1442 non-perfusion centres only group, n=1138 mixed perfusion centres only group, [∂] Late window: N=39 perfusion centres only group, n=375 non-perfusion centres only group, n=535 mixed perfusion centres only group

Table S7: Table of characteristics and outcomes according to time from stroke onset or last known well to endovascular treatment among patients selected **with or without futile recanalization** in the **early (<6 hours) and late (6-24 hours) time windows**.

Feature	Early window			Late window		
	**Futile n (%) median (IQR)**	**Non-Futile n (%) median (IQR)**	**P value**	**Futile n (%) median (IQR)**	**Non-Futile n (%) median (IQR)**	**P value**
Sample size	1203	1406		487	354	
Sex (male)	649 (53.9)	793 (56.4)	0.29	273 (56.0)	171 (48.3)	**0.026**
<60 years	230 (19.1)	437 (31.0)		141 (28.9)	120 (33..8)	
60-69	237 (19.7)	307 (21.8)		87 (17.8)	67 (18.9)	
70-79	398 (33.0)	404 (28.7)	**0.001**	139 (28.5)	106 (29.9)	0.26
80-89	285 (23.6)	239 (16.9)		106 (21.7)	57 (16.1)	
>90 years	53 (4.4)	19 (1.3)		14 (2.8)	4 (1.1)	
**Baseline characteristics**						
NIHSS on admission	19 (15-23)	16 (11-20)	**0.001**	17 (11-22)	12 (7-18)	**0.001**
Pre-stroke disability (mRS)	0 (0-1)	0 (0-0)	**0.001**	0 (0-1)	0 (0-0)	**0.029**
IV Thrombolysis	763 (63.4)	1054 (74.9)	0.001	165 (33.8)	110 (31.0)	0.39
Perfusion imaging use	205 (17.0)	291 (20.6)	0.018	171 (35.1)	147 (41.5)	0.058
Thromboaspiration[∂]	400 (36.0)	483 (37.6)	0.55	144 (31.8)	92 (28.1)	0.25
Stent-retriever[∂]	182 (16.3)	256 (19.9)	0.036	50 (11.0)	63 (19.2)	**0.002**
Thromboaspiration & Stent-retriever[∂]	529 (47.6)	544 (42.4)	**0.006**	258 (57.0)	172 (52.5)	0.20
Proximal Balloon Flow Arrest	259 (21.5)	298 (21.1)	0.83	130 (26.6)	95 (26.8)	0.96
**Co-morbidities**						
Hypertension	626 (52.0)	609 (43.8)	**0.001**	218 (44.7)	165 (46.6)	0.59
Diabetes	211 (17.5)	161 (11.3)	**0.001**	71 (14.5)	38 (10.7)	0.10
Atrial fibrillation	298 (24.7)	273 (19.9)	**0.001**	104 (21.3)	57 (16.1)	0.056
Prior Stroke/TIA	192 (15.9)	213 (15.3)	0.56	74 (15.1)	43 (12.1)	0.20
Congestive heart failure	63 (5.2)	70 (4.8)	0.76	28 (5.7)	19 (5.3)	0.81
**Outcomes**						
sICH	42/791 (5.3)	6/931 (0.6)	0.001	18/345 (5.2)	2/273 (0.7)	**0.004**
END	153/1150 (13.3)	28/1356 (2.0)	0.001	82/465 (17.6)	22/387 (6.1)	**0.001**
In-hospital mortality	257/1203 (21.3)	-	-	87/487 (17.8)	-	**-**

n = number of events, N = number of patients, mRS = modified Rankin scale, TIA = transient ischemic attack, NIHSS = National Institutes of Health Stroke Scale, IV = intravenous. Groups are compared using Chi-squared and Mann-Whitney U-tests as appropriate. [∂] Early window: N=1111 futile group, N= 1283 non-futile group, [∂] Late window: N=452 futile group, N= 327 non-futile group, Futile recanalization=mRS 4-6 at despite TICI2b-3 recanalization, sICH = symptomatic intracranial haemorrhage, END= Early neurological deterioration (NIHSS worsening by ≥4). * = statistically significant **adjusted multivariate analysis for age, sex, baseline NIHSS, pre-stroke disability, mode of anaesthesia, use of intravenous thrombolysis, centre, balloon guide catheter use and endovascular treatment technique.

Figure S1: Histogram demonstration of the number of patients (frequency) with time as a continuous variable in minutes across the early (< 6 hours; left) and late (6 to 24hours; right) endovascular thrombectomy time windows from stroke onset or last known well to arterial puncture.

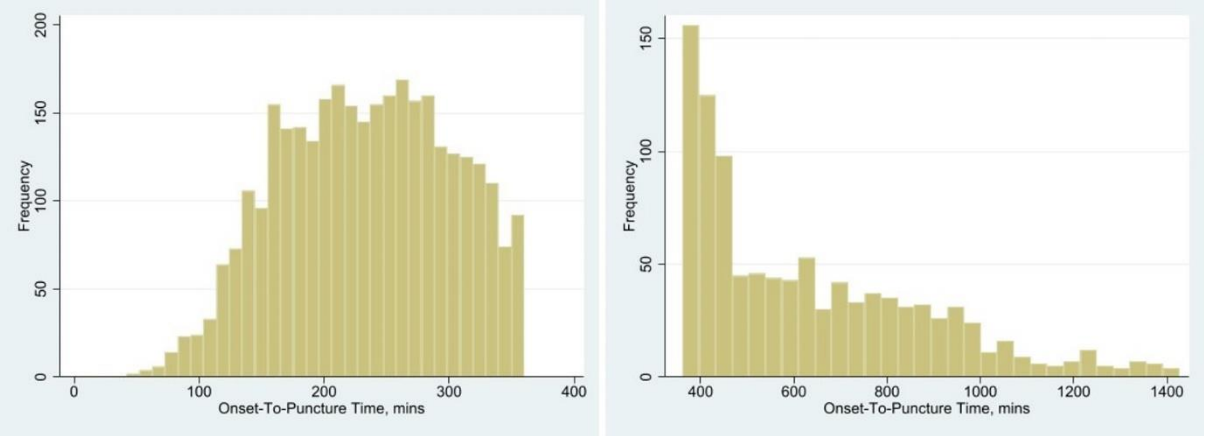

STROBE Statement—checklist of items that should be included in reports of observational studies

	Item No	Recommendation	Page No
**Title and abstract**	1	(*a*) Indicate the study's design with a commonly used term in the title or the abstract	1
		(*b*) Provide in the abstract an informative and balanced summary of what was done and what was found	4
**Introduction**			
Background/rationale	2	Explain the scientific background and rationale for the investigation being reported	6
Objectives	3	State specific objectives, including any prespecified hypotheses	6
**Methods**			
Study design	4	Present key elements of study design early in the paper	7
Setting	5	Describe the setting, locations, and relevant dates, including periods of recruitment, exposure, follow-up, and data collection	7
Participants	6	(*a*) *Cohort study*—Give the eligibility criteria, and the sources and methods of selection of participants. Describe methods of follow-up<br>*Case-control study*—Give the eligibility criteria, and the sources and methods of case ascertainment and control selection. Give the rationale for the choice of cases and controls<br>*Cross-sectional study*—Give the eligibility criteria, and the sources and methods of selection of participants	7
		(*b*) *Cohort study*—For matched studies, give matching criteria and number of exposed and unexposed<br>*Case-control study*—For matched studies, give matching criteria and the number of controls per case	7
Variables	7	Clearly define all outcomes, exposures, predictors, potential confounders, and effect modifiers. Give diagnostic criteria, if applicable	8-9
Data sources/ measurement	8*	For each variable of interest, give sources of data and details of methods of assessment (measurement). Describe comparability of assessment methods if there is more than one group	*8-9*
Bias	9	Describe any efforts to address potential sources of bias	9
Study size	10	Explain how the study size was arrived at	10
Quantitative variables	11	Explain how quantitative variables were handled in the analyses. If applicable, describe which groupings were chosen and why	9
Statistical methods	12	(*a*) Describe all statistical methods, including those used to control for confounding	9
		(*b*) Describe any methods used to examine subgroups and interactions	9
		(*c*) Explain how missing data were addressed	7
		(*d*) *Cohort study*—If applicable, explain how loss to follow-up was addressed<br>*Case-control study*—If applicable, explain how matching of cases and controls was addressed<br>*Cross-sectional study*—If applicable, describe analytical methods taking account of sampling strategy	9
		(*e*) Describe any sensitivity analyses	10

Continued on next page

**Results**			
Participants	13*	(a) Report numbers of individuals at each stage of study—eg numbers potentially eligible, examined for eligibility, confirmed eligible, included in the study, completing follow-up, and analysed	10-11
		(b) Give reasons for non-participation at each stage	10-11
		(c) Consider use of a flow diagram	10
Descriptive data	14*	(a) Give characteristics of study participants (eg demographic, clinical, social) and information on exposures and potential confounders	10-11
		(b) Indicate number of participants with missing data for each variable of interest	10-11
		(c) *Cohort study*—Summarise follow-up time (eg, average and total amount)	10-12
Outcome data	15*	*Cohort study*—Report numbers of outcome events or summary measures over time	*11*
		Case-control study—Report numbers in each exposure category, or summary measures of exposure	
		Cross-sectional study—Report numbers of outcome events or summary measures	
Main results	16	(*a*) Give unadjusted estimates and, if applicable, confounder-adjusted estimates and their precision (eg, 95% confidence interval). Make clear which confounders were adjusted for and why they were included	11-12
		(*b*) Report category boundaries when continuous variables were categorized	
		(*c*) If relevant, consider translating estimates of relative risk into absolute risk for a meaningful time period	
Other analyses	17	Report other analyses done—eg analyses of subgroups and interactions, and sensitivity analyses	12
**Discussion**			
Key results	18	Summarise key results with reference to study objectives	13
Limitations	19	Discuss limitations of the study, taking into account sources of potential bias or imprecision. Discuss both direction and magnitude of any potential bias	15-16
Interpretation	20	Give a cautious overall interpretation of results considering objectives, limitations, multiplicity of analyses, results from similar studies, and other relevant evidence	16
Generalisability	21	Discuss the generalisability (external validity) of the study results	15-17
**Other information**			
Funding	22	Give the source of funding and the role of the funders for the present study and, if applicable, for the original study on which the present article is based	17

*Give information separately for cases and controls in case-control studies and, if applicable, for exposed and unexposed groups in cohort and cross-sectional studies.

Note: An Explanation and Elaboration article discusses each checklist item and gives methodological background and published examples of transparent reporting. The STROBE checklist is best used in conjunction with this article (freely available on the Web sites of PLoS Medicine at http://www.plosmedicine.org/, Annals of Internal Medicine at http://www.annals.org/, and Epidemiology at http://www.epidem.com/). Information on the STROBE Initiative is available at www.strobe-statement.org.
